# Evaluation of Antiproliferative Potentials Associated with the Volatile Compounds of *Lantana camara* Flowers: Selective In Vitro Activity

**DOI:** 10.3390/molecules29225431

**Published:** 2024-11-18

**Authors:** Jennifer El Hajj, Louna Karam, Ali Jaber, Edmond Cheble, Elias Akoury, Philippe Hussein Kobeissy, José-Noel Ibrahim, Ali Yassin

**Affiliations:** 1 RDMPN Laboratory, Faculty of Pharmacy, Lebanese University, Beirut BP 14/6573, Lebanon; jenniferhajj0@gmail.com (J.E.H.); edmond.cheble@ul.edu.lb (E.C.); 2 Department of Biological Sciences, School of Arts and Sciences, Lebanese American University, Beirut 1102-2801, Lebanon; louna.karam@lau.edu.lb (L.K.); philippehussein.kobeissy@lau.edu.lb (P.H.K.); josenoel.ibrahim@lau.edu.lb (J.-N.I.); 3 Department of Physical Sciences, School of Arts and Sciences, Lebanese American University, Beirut 1102-2801, Lebanon; elias.akoury@lau.edu.lb

**Keywords:** bicyclogermacrene, *epi*-bicyclosesquiphellandrene, anticancer activity, *Lantana camara*, essential oil, chemical composition

## Abstract

Probing the chemical profiles and biological activities of medicinal plants is important for the discovery of new potent therapeutic products. Our study deciphers the chemical composition of the essential oils (EOs) obtained from three different flowers of *Lantana camara* and evaluates their antioxidant and anticancer activities. This work represents the first study of EOs obtained from this plant and is based particularly on the difference in flower color. In addition, no other reports dealing specifically with the antitumor effects of such flower-derived EOs have been described in the literature. The collected flowers, white, pink, and orange, were extracted by hydrodistillation to yield EO1, EO2, and EO3 respectively. Gas chromatography–mass spectroscopy was primarily employed to identify the existing volatile compounds in the samples. Their antioxidant activities were screened through both DPPH (2,2-diphenyl-1-picrylhydrazyl) scavenging assays and FRAP (ferric-reducing antioxidant power) assays. The antiproliferative effects were evaluated on two distinct breast cancer cell lines, MCF-7 and MDA-MB-231, and compared to a normal human breast cell line, MCF-10A, using an MTT (3-(4,5-dimethylthiazol-2-yl)-2,5-diphenyltetrazolium) assay. All EOs showed notable antioxidant potential attributed to the active phytochemical compounds, with results being supported by a positive correlation between such activity and the total phenolic and flavonoid content. The most eminent, EO1, revealed a selective dose-dependent antiproliferative effect in both breast cancer cell lines, thus reflecting its potent role as an anticancer agent. We suggest that this highly selective activity is associated with the presence of bicyclogermacrene and *epi*-bicyclosesquiphellandrene in its chemical composition.

## 1. Introduction

Known for its toxicity primarily due to the presence of lantadenes and other secondary metabolites [1], *Lantana camara* (*L. camara*) has a wide range of medicinal benefits [2] and biological activities [3,4]. In fact, this plant is one of the most noxious weeds in the world, while being toxic to animals and exerting allelopathic action on adjacent vegetation [5]. This dual-character perennial invasive shrub, originally native to the tropical regions of the Americas, is both an ornamental plant and a source of different bioactive compounds. Because of this, the careful analysis of its chemical composition within the scope of its biological effects has become of prime importance. On another note, the species is characterized by its vibrant and diverse flower colors, including mostly pink, white, and yellowish orange, as well as its small blackish fruits. Studies on the phytochemical profile of *L. camara* have shown a diverse array of secondary metabolites such as essential oils (EOs), phenols, flavonoids, alkaloids, glycosides, saponins, and tannins [6]. Historically, the plant has been used in folk medicine since ancient times, and correspondingly, antioxidant, antimicrobial [7], antipyretic, and insecticidal [8] effects have been reported by several groups recently in plant samples sourced from different geographical locations. Studies have shown significant discrepancies in the chemical compositions of the different extracts used to investigate *L. camara*’s biological activity [9]. While EOs are known for their complex mixtures of volatile compounds that contribute to their distinctive aroma and biological activities [6], their extraction and analysis have become crucial in identifying new bioactive compounds with potential therapeutic applications [10]. Comparably, some synthetic lantadenes which are pentacyclic triterpenoid derivatives obtained from the leaves of *L. camara* have been previously studied for their in vivo tumor inhibitory potential by Sharma et al. [11,12], while other groups presented some preliminary investigations on the use of *L. camara* stem extracts as inhibitors for leukemia cancer cells [13] or tried to investigate the underlying mechanism of its anticancer effects [14]. This is particularly important as cancer is becoming a major global health issue, ranking as the second leading cause of death after cardiovascular diseases. It is marked by the uncontrolled growth of cells, leading to malignant tumors with the potential to spread throughout the body [15,16,17]. Typical treatments have involved chemotherapy, radiotherapy, cytotoxic drugs, and surgery [18,19]. While these methods have shown success in treating various cancer types such as colon, pancreatic, testicular, breast, ovarian, and certain lung cancers, their overall effectiveness is often compromised by drug resistance and harmful side effects [20,21], which occur when healthy cells are unintentionally damaged during treatment. This underscores the need for ongoing research into safer, more effective treatments [8]. Interestingly, herbal medicines have been used for centuries in many developing countries and continue to serve as a primary form of healthcare [22]. Research has demonstrated that plants offer a promising source for the development of new anticancer drugs that are both effective and safe [23].

As a side note, gas chromatography–mass spectroscopy (GC-MS) is a powerful analytical technique used to identify and quantify the components of EOs, providing insights into their chemical profiles. In this study, we explored and analyzed the chemical compositions of EOs extracted from Lebanese *L. camara* flowers, more specifically from white, pink, and orange varieties. We then assessed their antioxidant activities as well as their antiproliferative effects on two different breast cancer cell lines. To the best of our knowledge, this work represents the first study of EOs obtained from *L. camara* based on different flower colors. Moreover, we are not aware of any reports dealing specifically with the antitumor effects of such flower-derived EOs. The aim of our study was to elucidate a potential correlation between chemical composition and the antitumor activity of EO, with the goal of advancing the development of new natural products with targeted therapeutic potential and possible clinical applications.

## 2. Results and Discussion

### 2.1. GC-MS Analysis Results and Yields of Extractions

**Figure 1:**
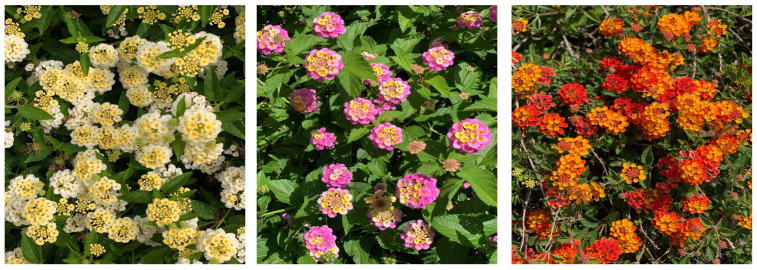
The three flowers of *L. camara* (white, pink, and orange) as collected.

Three different types of petals, namely, white, pink, and orange flowers from *L. camara* (Figure 1), were collected, dried, ground, and further extracted by hydrodistillation to yield the corresponding essential oils termed EO1, EO2, and EO3, respectively.

**Figure 2:**
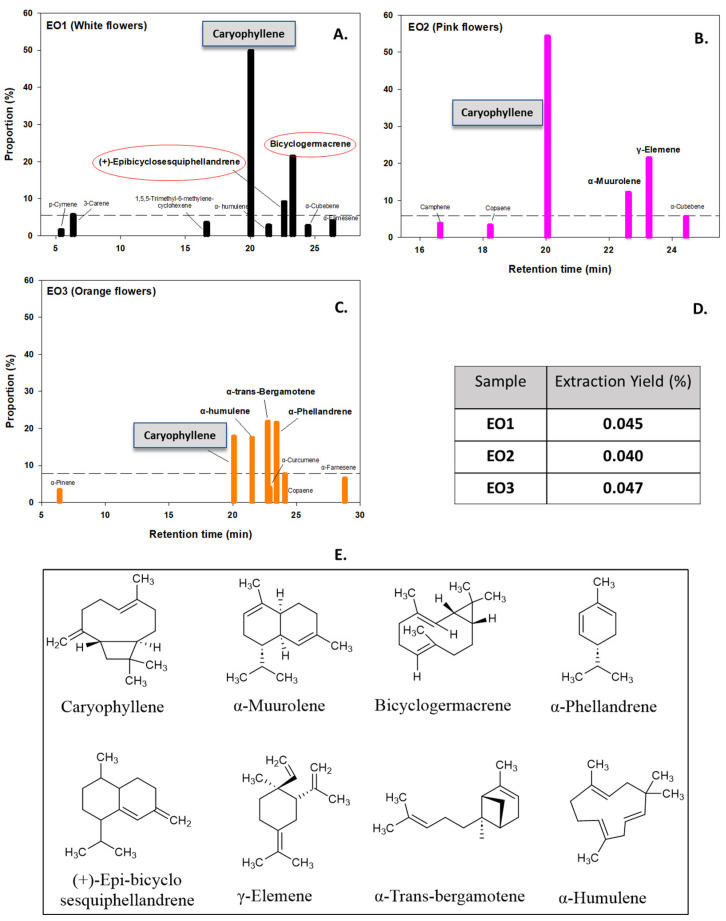
(**A**–**C**): Main constituents of the EOs of *L. camara* obtained from GC-MS analysis. (**D**): Yields of extraction of the three samples. (**E**): Chemical structures of the different compounds.

GC-MS analysis revealed that a major component of the three oils is caryophyllene, more precisely (−)-β-caryophyllene (BCP), a natural sesquiterpene which is also a cannabis-derived compound known to bind directly to endocannabinoid receptors in the body (Figure 2). Many studies have already reported caryophyllene as being a major constituent [9,24,25], while a more recent study pointed out that this chemical makes up ~70% of the EO of *L. camara* collected from India [8]. EO1 (white flowers) also contained bicyclogermacrene and (+)-*epi*-bicyclosesquiphellandrene at 21.34% and 9.04%, respectively. For EO2 (pink flowers), γ-elemene and α-muurolene were the other main components, while EO3 (orange flowers) was dominated by α-humulene, α-trans-bergamotene, and α-phellandrene, along with the presence of BCP. The results of the GC-MS presented in Figure 2 (additional details are given in Supplementary Materials) show that monoterpenes and sesquiterpenes are the sole components of the EOs of the collected flowers of *L. camara*. Such chemical compositions with profiles differing between each sample can explain the varying antioxidant and antiproliferative activities that will be presented herein. It is worth noting that β-caryophyllene is reported in the literature to have several biological activities such as antimicrobial and anti-inflammatory, but most notably, it functions as an antioxidant [26]. Other compounds like bicyclogermacrene, (+)-*epi*-bicyclosesquiphellandrene, γ-elemene, α-muurolene, α-humulene, and α-trans-bergamotene are sesquiterpenes that have been linked to cytotoxic, antimicrobial, antitumor, and many other potential activities [27,28].

Compared to other studies on EOs of *L. camara*, our findings highlight the critical difference in chemical composition based on geographic origin/location. For example, in Egypt, α-curcumene (10.26%), β-copene (12.29%), davanone (23.27%), caryophyllene (22.96%), and humulene (14.32%) are the major components [29]. A non-exhaustive table that attempts to compile the major compounds in the EOs from different studies can be found in the Supplementary Information. Similarly, considering the yields of extraction, a study in Brazil showed that there are significant differences between extraction yields obtained even at different collection times [30]. The lowest yield (0.01%) was recorded at 7:00 a.m., and the highest one (0.09%) was obtained at 7:00 p.m. In another study, the yield of an oil obtained in India was found to be 0.032% (*w*/*w*) [31]. In Africa, one group reported that the yield of EO of *L. camara* ranged from 0.25 to 0.37% *w*/*w* [32]. Our obtained yields are slightly above 0.04%, which is a similar value to those obtained in Brazil and in India. In general, yields of EOs are very low in most studies and vary according to the extraction method, time of collection, climate, seasonal time, and geographic origins.

### 2.2. Total Flavonoid Content (TFC) and Total Phenol Content (TPC)

It is known that EOs do not often contain flavonoids, as here, indeed, an extremely low content between 2 and 5 mg per 1 g of EO is observed. However, several reports in the literature have already indicated that some phenolic contents can be effectively extracted with essential oil [33,34]. In fact, the presence of phenolic terpenes or hydroxyl groups leads to the overestimation of the spectrophotometric test for *TFC* [35]. Meanwhile, the reason why flavonoids are not detected by GC-MS is due to the fact that they need derivatization in the sample preparation and the pre-injection step [36]. The analysis performed for *TFC* and TPC (as described in Section 3.4 and Section 3.5, and further in the Supplementary Materials) showed that EO3 from orange flowers had the greatest amount of flavonoids at 5.12 mg of rutin equivalent RE per 1 g of extract, while EO1 and EO2, from white and pink flowers, respectively, had contents of less than 3 mg of RE.g^−1^ (Table 1).

EO3 had also the highest amount of phenol content at 26.71 mg of GAE/g extract. EO2 showed a slightly lower TPC (24.63 mg of GAE/g extract), while that of EO1 was considerably low at 16.80 mg of GAE/g of extract (Table 1). A study reported in Madagascar revealed that the TPC of the flowers’ EO was 43.50 mg GAE/g of dry weight [37], while another study conducted in Nepal showed the highest phenol content to be 10.20 ± 0.34 mg GAE/g extract [38]. These findings further support our conclusion on the vast variability in phytochemical contents with regard to the geographical location of this flowering plant species, in addition to other factors including the extraction method and the storage procedure.

### 2.3. Antioxidant Activities

**Figure 3:**
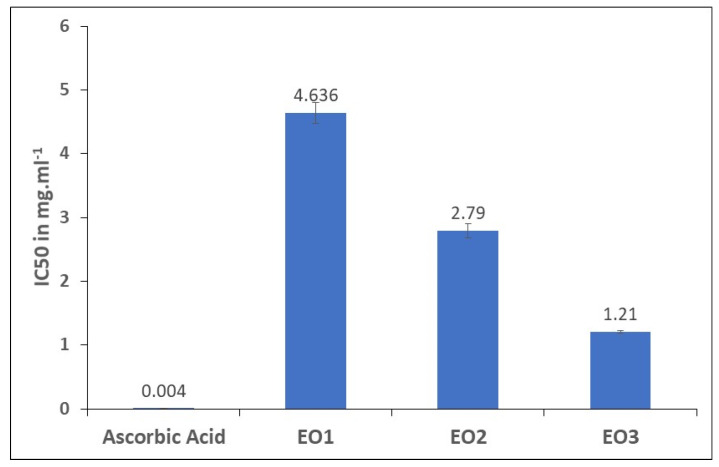
Results of IC50 of the three EOs as compared to ascorbic acid.

Given the fact that TPC and *TFC* are reported to be highly coupled to antioxidant capacity and are considered sources of natural antioxidants [39], we evaluated the in vitro antioxidant activities of the three EOs by both the 2,2-diphenyl-1-picrylhydrazyl (DPPH) assay and the ferric-reducing antioxidant power (FRAP) assay. Results obtained from the two tests were found to be dose-dependent. EO3 had the most potent antioxidant activity marked by its lowest IC_50_ = 1.21 mg.mL^−1^, followed by EO2 and EO1, which presented IC_50_ values of 2.79 mg.mL^−1^ and 4.64 mg.mL^−1^, respectively (Table 1). Nevertheless, all three EOs had much lower antioxidant activity than that of ascorbic acid with IC_50_ = 0.004 mg.mL^−1^ (Figure 3). According to one study conducted in Egypt, IC_50_ values were found to be 55.43 and 48.36 µg.mL^−1^ for the EOs of leaves and flowers, respectively [40]. In another study carried out in Pakistan, the DPPH (IC_50_) value was found to be 5.45 μg.mL^−1^ [41], while in Malaysia, the fruit extract showed an IC_50_ of 90.11 µg.mL^−1^ [42].

DPPH results were further validated by the FRAP assay, whereby EO3 indeed showed the highest antioxidant potential, and EO1 showed the lowest (Figure S10 in Supplementary Materials). This supports the reduction potential of the EOs transforming Fe^3+^ to Fe^2+^, and thus, their electron-donating ability. The differences in activity reported in the current work, and in the literature, can be ascribed to various factors. The difference in the chemical compositions of each sample is the most important among such factors. The higher antioxidant activity in EO3 is mostly due to the presence of trans-α-bergamotene, which is the predominant compound in this sample. The latter is a derivative of bergamotene, which has been shown to possess diverse biological activities such as antioxidant, anti-inflammatory, immunosuppressive, cytotoxic, antimicrobial, antidiabetic, and insecticidal effects [43]. The other major derivative, α-phellandrene, is known for its antioxidant and antibacterial activities, as well as being effective against liver cancer. Both molecules are not available in the other EOs, which may explain the potency of EO3 as an antioxidant [44]. In general, sesquiterpenes are reported to be responsible for several biological activities [45], a fact that can elucidate their high antioxidant effect. As a side note, differences in IC_50_ values can also be due to ecological factors such as the age of the plant, humidity, height, temperature, and water, all which influence the quality and the quantity of the secondary metabolites, which are responsible for different biological activities, such as the phenols, flavonoids [46], monoterpenes, and sesquiterpenes that are present in the EO of *L. camara*.

### 2.4. Reduction in MCF-7 and MDA-MB-231 Cell Proliferation in a Dose-Dependent Manner

**Figure 4:**
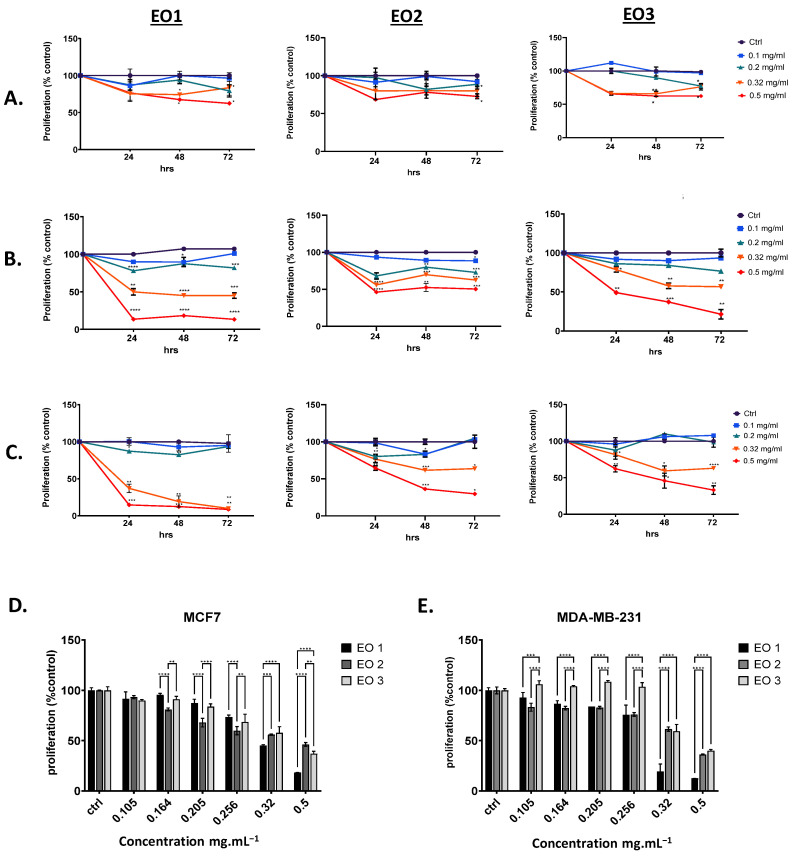
In vitro cytotoxic effect of EO1, EO2, and EO3 on the MCF-10A, MCF-7, and MDA-MB231 cell lines. Representative graphs of the time-dependent cytotoxic effect of different concentrations of the oils on MCF-10A (**A**), MCF-7 (**B**), and MDA-MB-231 (**C**) cells. Dose-dependent inhibitory activity for EO1, EO2, and EO3 48 h post-treatment on MCF-7 (**D**) and MDA-MB-231 (**E**) is presented as a bar plot. Data are presented as mean ± SD (n = 3) with all treatments compared to each other by a two-way ANOVA test followed by the post hoc Tukey test for multiple comparison analysis; * *p* < 0.05, ** *p* < 0.01, *** *p* < 0.001, **** *p* < 0.0001.

Antitumor activities were explored on two different breast cancer (BC) cell lines: MCF-7, a non-invasive ER+ PR+ cell line, and MDA-MB-231, an invasive triple negative BC cell line. At the same time, MCF-10A, an immortalized epithelial breast cell line, was used to assess toxicity in normal cells. As shown in Figure 4, both cancer cell lines displayed dose-dependent sensitivities to increasing concentrations of the three tested EOs. Interestingly, EO1 from white flowers exhibited the strongest antiproliferative effect on both cell lines 24 h post-treatment. The calculation of the IC_50_ revealed an approximate value of 0.3 g.mL^−1^ in both cell lines and at all time-points. EO2 was significantly less potent in inhibiting proliferation, particularly in MCF7, where cell proliferation remained greater than 50% even 72 h post-treatment, while EO3 demonstrated intermediate inhibitory potential compared to the other samples. Though EO3 showed greater potency than EO2, both were still significantly less effective than EO1, in particular at higher concentrations. Cytotoxicities are detailed in Figure 4, with dose-dependent inhibitory activities presented as a bar plot for each BC cell line. The results were further supported by the higher IC_50_ values for EO2 and EO3 at different time-points, as shown in Table 2.

More importantly, all three EOs had a less significant cytotoxic effect on the normal human breast cell line, MCF-10A, proliferation, where the inhibitory effect was only noted when cells were treated with very high concentrations, up to 0.5 mg.mL^−1^. The calculation of the IC_50_ revealed estimated values higher than 1 mg.mL^−1^, which are at least 2–3 times greater than those observed in MCF-7 and MDA-MB-231 cells (Table 2), hence indicating the greater selectivity of these natural compounds against cancer cells.

Our findings conclude that the EO isolated from the white flowers of *L. camara* demonstrates selective antitumor activity against breast cancer, while showing no significant antiproliferative effect on normal cells. To the best of our knowledge, no study to date has explored the antitumor potential of an EO obtained from *L. camara* flowers. EOs from the leaves, however, have been shown to account for strong anticancer activity against U-266, A-549, HCT-116, SCC-4, MiaPaCa 2, and KBM-5 cancer cell lines [39].

**Figure 5:**
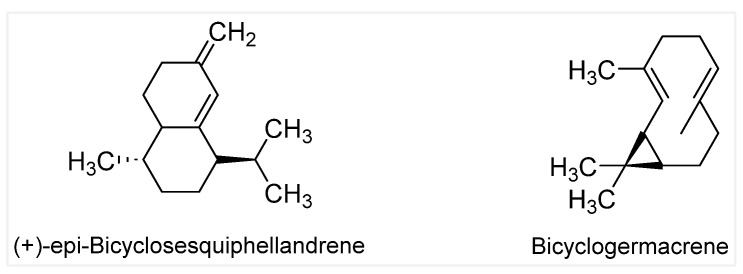
Chemical structures of the possible antitumor-active phytochemicals in EO1.

The differences in growth inhibitory effect could be explained by the results of GC-MS analysis which revealed distinct chemical profiles for each sample. We attribute the antiproliferative activity of EO1 against the two BC cell lines reported herein to the presence of *epi*-bicyclosesquiphellandrene (Figure 5) found exclusively, and in significant proportions, in the EO of white flowers. A study on the oily fractions of Teucrium alopecurus showed that the *epi*-bicyclosesquiphellandrene molecule had antitumor activity against colon cancer cells [47]. Another major component present exclusively in EO1 is bicyclogermacrene, which can also contribute to the significant antitumoral activity. Indeed, one research paper conducted in Brazil has shown that bicyclogermacrene plays a major role in the anticancer activity of the Myrcia genus plant against lung cancer cells [48].

### 2.5. Pearson’s Correlation Coefficient Analysis

The relationship between the TPC and *TFC* present in the EOs, and their in vitro antioxidant and antitumor activities, was expressed by adopting Pearson’s correlation coefficients (PCCs), also referred to as Pearson’s r, which are represented in Table 3.

The PCCs of −0.9260 and −0.9617 imply a strong negative correlation between *TFC*/DPPH and TPC/DPPH, respectively. In general, a sample with high levels of *TFC* and TPC gives a high level of DPPH where phenolic compounds are classified according to the hydroxyl groups attached to the benzene ring. Such hydroxyl groups are usually good hydrogen donors and can react and neutralize reactive oxygen such as DPPH [49,50]. Consequently, a high concentration of phenolic compounds in an extract is often followed by high antioxidant activity. On the other hand, the IC_50_ values of MCF-7, at 24 h post-treatment, showed an extremely high positive correlation to the total flavonoid content (correlation coefficient: 0.99997), with a strong negative correlation with the IC_50_ of DPPH (correlation coefficient: −0.9286). Based on such findings, two conclusions can be suggested: First, flavonoids seem to be an important contributor to the antitumor effect against MCF-7, and, second, the radical scavenging mechanism follows a highly similar mechanism through which the antitumor effect against MCF-7 cells takes place. We note similarly that the content of phenols shows a strong positive correlation (0.9186) with the antitumor effect over MDA-MB-231 and its IC_50_ value at 24 h post-treatment.

## 3. Materials and Methods

### 3.1. Collection of the Petals of L. camara

Three different flower colors of L. camara were freshly collected from the Lebanese University at Hadath, Beirut (33°49′39″ N, 35°31′17″ E) during June and July 2023. Every time, 100 to 150 g of fresh white, pink, or orange petals was collected and divided into three flasks of 1 L capacity in order to perform the extraction process.

### 3.2. Hydrodistillation with Clevenger

Ground flowers of *L. camara* were subjected to hydrodistillation with the Clevenger apparatus under optimal operational conditions. First, 40 g of the flowers was mixed with 400 mL of distilled water. The distillation process was performed for 3 h, and the obtained essential oil was collected and dehydrated using anhydrous Na_2_SO_4_.

### 3.3. GC-MS Analysis

GC-MS analysis was carried out using the electron ionization method. The GC capillary column used was an Agilent 19091S-433 (Agilent Technologies, Santa Clara, CA, USA), HP-5MS with 5% phenyl methyl siloxane, film thickness of 0.25 μm, a length of 30 m, and an internal diameter of 250 µm. Helium was used as the carrier gas with a column head pressure of 1.09 bar, flow rate of 1 mL.min^−^^1^, and 1 μL injections in split mode (1:50). The initial column temperature applied was 65 °C, which then increased to reach 450 °C. The GC oven temperature program ranged between an initial temperature of 65 °C and final temperature of 200 °C, with a run time of 45 min. The mass spectrometer (Agilent Technologies, Santa Clara, CA, USA) was operated in the EI mode at 70 eV with a mass scanning range of 50–500 and a source temperature of 230 °C. The identity of each compound was determined based on their retention indices and by comparison of their mass spectral fragmentation patterns with those reported in the NIST library database (NIST). The quantitative analysis expressing the percentage of the identified components in each volatile oil was obtained by the integration of the peak areas. Only fully identified compounds are reported in this study.

### 3.4. TFC Analysis

*TFC* was determined by the aluminum chloride colorimetric method [51]. In a test tube, 1 mL of the diluted plant extract solution and 1 mL of AlCl_3_.6H_2_O (2% solution) were mixed together and left in the dark at room temperature for 30 min to react. After incubation, the absorbance of the developed yellow color was measured at λ_max_ = 410 nm using a double-beam UV-VIS spectrophotometer against a blank solution. The same procedure was carried out using rutin as a reference standard (0–0.09 mg.mL^−^^1^). A standard curve of absorbance versus different concentrations of rutin was plotted. Results were reproduced in triplicate for each analytical trial, from which the mean and standard deviation values were calculated [52]. *TFC* was determined from the linear equation of a standard curve prepared with rutin and expressed in mg of rutin equivalent (RE) per g of plant extract using the following equation:

$$TFC=\frac{C\times V\times DF}{m}$$

where *C* is the concentration of rutin calculated by the calibration curve regression equation in mg.mL^−^^1^, *V* is the volume of plant extract solution in mL, *DF* is the dilution factor, and *m* is the mass of extract in g used to prepare the plant extract solution.

### 3.5. TPC Analysis

TPC was determined by the Folin-Ciocalteu reagent (FCR) method. In this procedure, 100 μL of the diluted plant extract was added to 500 µL of FCR and incubated for 5 min in the dark. Then, 2 mL of Na_2_CO_3_ was added and samples were shaken and left in the dark at room temperature for 30 min. The absorbance of the developed blue color was measured at 760 nm using a UV-6300PC double-beam Spectrophotometer (VWR, Leuven, Belgium) against a blank solution. The same procedure was carried out using gallic acid as a reference standard (0–0.27 mg.mL^−^^1^); then, a standard curve of absorbance versus different concentrations of gallic acid was plotted [53]. The TPC was determined from the linear equation of a standard curve prepared with gallic acid and expressed in mg gallic acid equivalent (GAE) per g of plant extract using the following equation:

$$x=\frac{C\times V\times DF}{m}$$

where *C* is the concentration of gallic acid calculated by the calibration curve regression equation in mg.mL^−^^1^, *V* is the volume of plant extract solution in mL, *DF* is the dilution factor, and *m* is the mass of extract in g used to prepare the plant extract solution.

### 3.6. DPPH Assay

The free radical scavenging activity of the three samples and of the ascorbic acid solution was investigated using the DPPH method [54]. In this procedure, 1 mL of the PE solution of different concentrations was added to 1 mL of the DPPH methanolic solution. A control consisting of 1 mL of DPPH solution with 1 mL methanol was also prepared. The mixtures were shaken vigorously and then incubated in the dark at room temperature for 30 min to reach a steady state. The color change, from violet at a low concentration to yellow at a high concentration, was determined by measuring the absorbance at 520 nm against a blank solution using a double-beam UV-VIS spectrophotometer. The same procedure was carried out for ascorbic acid, a pure antioxidant compound, which was used as a standard reference [55]. The percentage of the scavenging activity of each extract on the DPPH radical was calculated as % inhibition of DPPH using the following equation:

$$\%\;scavenging=\frac{A\;(control)-A\;(sample)}{A\;control\;}\times 100\;$$

where *A* (*control*) is the absorbance of DPPH alone, and *A* (*sample*) is the absorbance of DPPH with different concentrations of extracts. The concentration of the extract required to scavenge 50% of the DPPH free radical (IC_50_) was determined from the percentage curve of DPPH inhibitions versus extract concentration. The antioxidant activity of all samples and their IC_50_ was compared to that of ascorbic acid.

### 3.7. FRAP Assay

A serial dilution of extract solutions (0.35–2.1 mg.mL^−^^1^) and ascorbic acid (0.015–0.15 mg.mL^−^^1^) was prepared in ethanol. First, 200 μL of each extract solution was mixed with 200 μL of 0.2 M phosphate buffer (pH 6.6) and 200 μL of potassium ferricyanide (1%). The reaction mixtures were incubated at 50 °C for 20 min. After cooling, 200 μL of trichloroacetic acid (10%) was added and the mixtures were centrifuged at 1000 rpm for 8 min. The upper layer (800 μL) was mixed with 800 μL of distilled water and 160 μL of ferric chloride (0.1%). After a 10 min reaction time, the spectrometric absorbance was recorded at 700 nm and compared with ascorbic acid as the positive control. The absorbance values were plotted against the concentration, and a linear regression analysis was carried out. Higher absorbance readings indicate higher reducing power [56].

### 3.8. Cell Culture

The human breast cancer MCF-7 and MDA-MB-231 cell lines were purchased from the American Type Culture Collection (ATCC, Manassas, VA, USA). MCF-7 and MDA-MB-231 were cultured as previously described [57,58]. MCF-10A cells were cultured in DMEM-F12 supplemented with horse serum (5%), hydrocortisone (0.5 μg.mL^−^^1^), EGF (20 ng.mL^−^^1^), insulin (10 μg.mL^−^^1^), 100 U.mL^−^^1^ of penicillin, and 100 μg.mL^−^^1^ of streptomycin. Cells were either left untreated (control) or treated with different concentrations of EOs for 24, 48, and 72 h.

### 3.9. Cell Proliferation Assay

The antiproliferative effect of the EOs was assessed using the MTT (3-(4,5-dimethylthiazol-2-yl)-2,5-diphenyltetrazolium bromide) assay (Sigma-Aldrich, Darmstadt, Germany). This assay determines the cell viability by measuring the amount of formazan dye that is produced upon cellular reduction in MTT by metabolically active cells. MCF-7, MDA-MB-231, and MCF-10A cells were seeded in triplicate for each condition in 96-well plates at a density of 7 × 10^3^ cells/0.1 mL, 5 × 10^3^ cells/0.1 mL, and 10 × 10^3^ cells/0.1 mL respectively. After adherence, cells were left untreated or treated with a concentration range of 0–500 mg.mL^−^^1^ of EO1, EO2, or EO3 for 24, 48, and 72 h. At each time-point, 10 μL/well of MTT reagent (5 mg.mL^−^^1^) was added, and cells were incubated at 37 °C for 3h. The reaction was stopped and the formazan crystals were dissolved by adding 100 μL of 10% SDS in 0.01 N HCl. Absorbance was measured at 570 nm using a Varioskan Flash plate reader (Thermo Fisher Scientific, Waltham, MA, USA). Cell proliferation was represented as the mean percent control plotted against the log of concentration and incubation time of three independent experiments repeated for each sample. IC_50_ values were generated from the non-linear regression with variable-slope dose-response curves.

### 3.10. Statistical Analyses

Statistical analyses were performed using the GraphPad Prism 5.04 software (GraphPad, San Diego, CA, USA). Values are expressed as mean ± SD (standard deviation). Differences between control and treated groups were assessed for statistical significance by the two-way ANOVA test followed by the post hoc Tukey test for multiple comparison analysis. Each experiment was repeated at least three times, and each condition was carried out in triplicate.

## 4. Conclusions and Outlook

In a nutshell, we are reporting on the first study of EOs obtained from *L. camara* flowers based on their distinct blossom color. The work deals specifically with the antitumor effect of such flower-derived samples. GC-MS analysis revealed different chemical profiles for the EOs of white, pink, and orange flowers. EO1 (white flowers) was shown to be distinctively rich in (+)-*epi*-bicyclosesquiphellandrene and bicyclogermacrene, both of which are known for their antitumor activities. Interestingly, EO3 (orange flowers) showed the highest antioxidant activity in comparison to a very low performance for EO1. On the other hand, the highly potent anticancer activity of the latter was perceived on MCF-7 and MDA-MB-231 breast cancer cell lines. EO1 exhibited such strong activity by significantly inhibiting the cell proliferation at lower concentrations, while selectively targeting cancer cells over normal breast cells. This study underscores the importance of exploring the detailed chemical diversity of plant-derived essential oils in relation to their biological activities, paving the way for the discovery of new bioactive compounds with potential clinical applications. However, further research is needed to isolate and characterize the specific active compounds and to elucidate their mechanisms of action in cancer therapy. Additionally, we are interested in the evaluation of the antioxidant and antitumor effects of some commercially available compounds that are components of the three EOs and that could be tested in their pure form.

## Figures and Tables

**Table 1:** Results of the *TFC*, TPC, and IC50 of the three sample with a dilution factor *DF* = 1.

Sample	*TFC* (mg of RE/g of Extract)	TPC (mg of GAE/g of Extract)	IC_50_ (mg/mL) ^1^
EO1	2.41	16.80	4.64
EO2	2.93	24.63	2.79
EO3	5.12	26.71	1.21

^1^ Ascorbic acid value = 0.004.

**Table 2:** Mean IC_50_ values of EOs on MCF-7, MDA-MB-231, and MCF-10A cell lines at 24-, 48-, and 72 h post-treatment.

Cell Line	Time (h)	IC_50_ (g.mL^−1^)		
		EO1	EO2	EO3
MCF-7 (BC)	24	0.3061 ± 0.091	0.3630 ± 0.057	0.5927 ± 0.03
	48	0.3021 ± 0.043	0.4101 ± 0.109	0.3937 ± 0.09
	72	0.3179 ± 0.002	0.4144 ± 0.09	0.3397 ± 0.027
MDA-MB-231 (BC)	24	0.3099 ± 0.001	0.7398 ± 0.012	0.6473 ± 0.005
	48	0.2820 ± 0.022	0.4015 ± 0.01	0.4150 ± 0.06
	72	0.2800 ± 0.023	0.3931 ± 0.058	0.3951 ± 0.08
MCF-10A (normal)	24	>1	>1	>1
	48	>1	>1	>1
	72	>1	>1	>1

**Table 3:** Pearson’s correlation coefficients for *TFC*, TPC, DPPH, MCF-7, and MDAMB-231 in the three EOs under consideration.

	*TFC*	TPC	DPPH	MCF-7	MDA-MB-231 *
*TFC*	1				
TPC	0.7869	1			
DPPH	−0.9260	−0.9617	1		
MCF-7	0.99997	0.7913	−0.9286	1	
MDA-MB-231	0.4790	0.9186	−0.7750	0.4853	1

* Relevant IC_50_ values at 24 h post-treatment.

## Data Availability

The original contributions presented in the study are included in the article/Supplementary Materials; further inquiries can be directed to the corresponding authors.

## Supplementary Materials

The following supporting information can be downloaded at https://www.mdpi.com/article/10.3390/molecules29225431/s1, Figure S1: Petals of the *Lantana Camara* flowers: White, Pink, and Orange, respectively; Table S1: Main constituents of *L. Camara* white flowers essential oil (EO1); Figure S2: GC-MS chromatogram of EO1; Table S2: Main constituents of *L. Camara* pink flowers essential oil (EO2); Figure S3: GC-MS chromatogram of EO2; Table S3: Main constituents of *L. Camara* orange flowers essential oil (EO3); Figure S4: GC-MS chromatogram of EO3; Table S4: Main constituents of EOs of *Lantana Camara* from different geographical origins previously reported in literature; Table S5: Results of the phytochemical screening of *L. Camara* flowers; Figure S5: Variation of the Absorbance in function of the concentration of the Rutin in µg/mL; Table S6: Results of the *TFC* of the different samples; Figure S6: Comparison of the *TFC* in different extracts of *Lantana Camara*; Figure S7: Variation of the Absorbance in function of the concentration of Gallic Acid in mg/mL; Table S7: Results of TPC of the different samples; Figure S8: Comparison of the TPC in different essential oil extracts of *Lantana Camara*; Figure S9: Results of the DPPH scavenging assay in the three EOs and the ascorbic acid measuring the % Scavenging in function of its different concentrations in mg/mL; Table S8: Results of DPPH Assay of the different samples; Figure S10: Results of the FRAP assay of the three EO samples, measuring absorbance as function of the different concentrations in mg.mL^−1^ (see [9,29,40,59,60,61]).

## Abbreviations

The following is a list of abbreviations in the sequence of their appearance. EO: essential oils; DPPH: 2,2-diphenyl-1-picrylhydrazyl; FRAP: ferric-reducing antioxidant power; MTT: 3-(4,5-dimethylthiazol-2-yl)-2,5-diphenyltetrazolium; *L. camara*: *Lantana camara*; GC-MS: gas chromatography–mass spectroscopy; BCP: (−)-β-caryophyllene; *TFC*: total flavonoid content; TPC: Total Phenol Content; BC: breast cancer; PCC: Pearson’s correlation coefficients; RE: rutin equivalent; FCR: Folin–Ciocalteu reagent; GAE: gallic acid equivalent; ATCC: American Type Culture Collection; SD: standard deviation.

## References

[1] Parimoo H.A., Sharma R., Patil R.D., Patial V. (2015). Sub-acute toxicity of lantadenes isolated from *Lantana camara* leaves in guinea pig animal model. Comp. Clin. Pathol..

[2] Kalita S., Kumar G., Karthik L., Rao K.V.B. (2012). A Review on Medicinal Properties of *Lantana camara* Linn. Res. J. Pharm. Technol..

[3] Nayak B.S., Raju S.S., Eversley M., Ramsubhag A. (2009). Evaluation of wound healing activity of *Lantana camara* L.—A Preclinical study. Phytother. Res..

[4] Ganesh T., Saikat S., Thilagam E., Thamotharan G., Loganathan T., Chakraborty R. (2010). Pharmacognostic and anti-hyperglycemic evaluation of *Lantana camara* (L.) var. aculeate leaves in alloxan-induced hyperglycemic rats. Int. J. Res. Pharm. Sci..

[5] Sharma O.P., Makkar H.P.S., Dawra R.K. (1988). A review of the noxious plant *Lantana camara*. R.K. Toxicon.

[6] Shah M., Alharby H.F., Hakeem K.R. (2020). *Lantana camara*: A Comprehensive Review on Phytochemistry, Ethnopharmacology and Essential Oil Composition. Lett. Appl. NanoBioSci..

[7] Mansoori A., Singh N., Dubey S.K., Thakur T.K., Alkan N., Das S.N., Kumar A. (2020). Phytochemical Characterization and Assessment of Crude Extracts From *Lantana camara* L. for Antioxidant and Antimicrobial Activity. Front. Agron..

[8] Aisha K., Visakh N.U., Pathrose B., Mori N., Baeshen R.S., Shawer R. (2024). Extraction, Chemical Composition and Insecticidal Activities of *Lantana camara* Linn. Leaf Essential Oils against *Tribolium castaneum*, *Lasioderma serricorne* and *Callosobruchus chinensis*. Molecules.

[9] Nea F., Kambiré D., Genva M., Tanoh E., Wognin E., Martin H., Brostaux Y., Tomi F., Lognay G.C., Tonzibo Z. (2020). Composition, Seasonal Variation, and Biological Activities of *Lantana camara* Essential Oils from Côte d’Ivoire. Molecules.

[10] El Kantar S., Yassin A., Nehmeh B., Labaki L., Mitri S., Naser Aldine F., Hirko A., Caballero S., Monck E., Garcia-Maruniak A. (2022). Deciphering the therapeutical potentials of rosmarinic acid. Sci. Rep..

[11] Sharma M., Sharma P.D., Bansal M.P., Singh J. (2007). Synthesis, Cytotoxicity, and Antitumor Activity of Lantadene-A Congeners. Chem. Biodivers..

[12] Sharma M., Sharma P.D., Bansal M.P. (2008). Lantadenes and Their Esters as Potential Antitumor Agents. J. Nat. Prod..

[13] Babar V.B., Khapale P.R., Nagarale S. (2019). Preliminary investigation and in-vitro anticancer activity of *Lantana camara* L. (Verbenaceae). J. Pharmacogn. Phytochem..

[14] Han E.B., Chang B.Y., Jung Y.S., Kim S.Y. (2015). *Lantana camara* Induces Apoptosis by Bcl-2 Family and Caspases Activation. Pathol. Oncol. Res..

[15] Wang H., Khor T.O., Shu L., Su Z.-Y., Fuentes F., Lee J.-H., Kong A.-N.T. (2012). Plants vs. Cancer: A Review on Natural Phytochemicals in Preventing and Treating Cancers and Their Druggability. Anti-Cancer Agents Med. Chem..

[16] Ochwang’i D., Kimwele C., Oduma J., Gathumbi P., Mbaria J., Kiama S.J. (2014). Medicinal plants used in treatment and management of cancer in Kakamega County, Kenya. J. Ethnopharmacol..

[17] Greenwell M., Rahman P.K.S.M. (2015). Medicinal Plants: Their Use in Anticancer Treatment. Int. J. Pharm. Sci. Res..

[18] Aiello P., Sharghi M., Mansourkhani S.M., Ardekan A.P., Jouybari L., Daraei N., Peiro K., Mohamadian S., Rezaei M., Heidari M. (2019). Medicinal Plants in the Prevention and Treatment of Colon Cancer. Oxidative Med. Cell. Longev..

[19] O’Reilly M.S., Boehm T., Shing Y., Fukai N., Vasios G., Lane W.S., Flynn E., Birkhead J.R., Olsen B.R., Folkman J. (1997). Endostatin: An Endogenous Inhibitor of Angiogenesis and Tumor Growth. Cell.

[20] The American Cancer Society Medical and Editorial Content Team: Chemotherapy Side Effects. https://www.cancer.org/cancer/managing-cancer/treatment-types/chemotherapy/chemotherapy-side-effects.html.

[21] Cragg G.M., Newman D.J. (2005). Plants as a source of anti-cancer agents. J. Ethnopharmacol..

[22] Sivaraj R., Rahman P., Rajiv P., Narendhran S., Venckatesh R. (2014). Biosynthesis and characterization of *Acalypha indica* mediated copper oxide nanoparticles and evaluation of its antimicrobial and anticancer activity. Spectrochim. Acta A Mol. Biomol. Spectrosc..

[23] Hassan B. (2020). Plants and Cancer Treatment. Medicinal Plants—Use in Prevention and Treatment of Diseases.

[24] Zoubiri S., Aoumeur B. (2012). Chemical Composition and Insecticidal Properties of *Lantana camara* L. Leaf Essential Oils from Algeria. J. Essent. Oil Res..

[25] Chowdhury J.U., Nandi N.C., Nazrul I., Bhuiyan N.I. (2007). Chemical composition of leaf essential oil of *Lantana camara* L. from Bangladesh. Bangladesh J. Bot..

[26] Gushiken L., Beserra F., Hussni M., Gonzaga M., Ribeiro V., Fernanda de Souza P., Campos J., Massaro T., Hussni C., Takahira R. (2022). Beta-caryophyllene as an antioxidant, anti-inflammatory and re-epithelialization activities in a rat skin wound excision model. Oxidative Med. Cell. Longev..

[27] Wang J.-H., Luan F., He X.-D., Wang Y., Li M.-X. (2018). Traditional uses and pharmacological properties of Clerodendrum phytochemicals. J. Tradit. Complement. Med..

[28] Do A.R., Emami S.A., Akaberi M. (2024). Phytochemical diversity and pharmacological effects of sesquiterpenes from *Artemisia* spp.. Phytochem. Rev..

[29] Sundufu A.J., Shoushan H. (2004). Chemical composition of the essential oils of *Lantana camara* L. occurring in south China. Flavour Fragr. J..

[30] Sousa E.O., Colares A.V., Rodrigues F.F.G., Campos A.R., Lima S.G., Costa J.G.M. (2010). Effect of Collection Time on Essential Oil Composition of *Lantana camara* Linn (Verbenaceae) Growing in Brazil Northeastern. Rec. Nat. Prod..

[31] Singh R.K., Tiwari B., Sharma U., Singh S.P. (2012). Chemical Composition of *Lantana camara* Fruit Essential Oil. Asian J. Chem..

[32] Liambila R., Wesonga J., Ngamau C., Wallyambillah W. (2021). Chemical composition and bioactivity of *Lantana camara* L. essential oils from diverse climatic zones of Kenya against leaf miner (*Tuta absoluta* Meyrick). Afr. J. Agric. Res..

[33] Mamadaliev A.N., Kushiev K.K., Abdullaeva Z.R. (2022). Determination of Flavonoids in Different Oils of *Cannabis sativa* L (Cannabaceae) and Evaluation of Physico-chemical Indicators. Austrian J. Tech. Nat. Sci..

[34] Semeniuc C.A., Socaciu M.-I., Socaci S.A., Mureșan V., Fogarasi M., Rotar A.M. (2018). Chemometric Comparison and Classification of Some Essential Oils Extracted from Plants Belonging to Apiaceae and Lamiaceae Families Based on Their Chemical Composition and Biological Activities. Molecules.

[35] Shraim A.M., Ahmed T.A., Rahman M., Hijji Y.M. (2021). Determination of total flavonoid content by aluminum chloride assay: A critical evaluation. LWT.

[36] Zhang K., Zuo Y.J. (2004). GC-MS Determination of Flavonoids and Phenolic and Benzoic Acids in Human Plasma after Consumption of Cranberry Juice. J. Agric. Food Chem..

[37] Al Snafi A.E. (2019). Chemical Constituents and Pharmacological Activities of *Lantana Camara*—A Review. Asian J. Pharm. Clin. Res..

[38] Kapali J., Sharma K.R. (2021). Estimation of phytochemicals, antioxidant, antidiabetic and brine shrimp lethality activities of some medicinal plants growing in Nepal. J. Med. Plants.

[39] Li M., Paré P.W., Zhang J., Kang T., Zhang Z., Yang D., Wang K., Xing H. (2018). Antioxidant Capacity Connection with Phenolic and Flavonoid Content in Chinese Medicinal Herbs. Rec. Nat. Prod..

[40] El Baroty G.S., Goda H.M., Khalifa E.A., Abd El Baky H.H. (2014). Antimicrobial and antioxidant activities of leaves and flowers essential oils of Egyptian *Lantana camara* L.. Der Pharma Chem..

[41] Sajid A., Manzoor Q., Imran M., Aslam F., Gondal T.A., Ahmad R.S., Hussain G., Imran M., Aslam F., Arshad M. (2021). Essential Oil and Leaves from *Lantana camara* Significantly Ameliorate Different Cancer Cell Lines by Suppressing the NF-κB Pathway. Sains Malays..

[42] Mahdi-Pour B., Jothy S.L., Latha L.Y., Chen Y., Sasidharan S. (2012). Antioxidant activity of methanol extracts of different parts of *Lantana camara*. Asian Pac. J. Trop. Biomed..

[43] Annaz H., El Fakhouri K., Ben Bakrim W., Mahdi I., El Bouhssini M., Sobeh M. (2024). Bergamotenes: A comprehensive compile of their natural occurrence, biosynthesis, toxicity, therapeutic merits and agricultural applications. Crit. Rev. Food Sci. Nutr..

[44] Radice M., Durofil A., Buzzi R., Baldini E., Martínez A.P., Scalvenzi L., Manfredini S. (2022). Alpha-Phellandrene and Alpha-Phellandrene-Rich Essential Oils: A Systematic Review of Biological Activities, Pharmaceutical and Food Applications. Life.

[45] Ivanescu B., Miron A., Corciova A.J. (2015). Sesquiterpene Lactones from *Artemisia Genus*: Biological Activities and Methods of Analysis. J. Anal. Methods Chem..

[46] Karak P. (2019). Biological Activities of Flavonoids: An Overview. Int. J. Pharm. Sci. Res..

[47] Guesmi F., Tyagi A.K., Prasad S., Landoulsi A. (2018). Terpenes from essential oils and hydrolate of *Teucrium alopecurus* triggered apoptotic events dependent on caspases activation and PARP cleavage in human colon cancer cells through decreased protein expressions. Oncotarget.

[48] Montalvão M.M., Felix F.B., Propheta dos Santos E.W., Santos J.F., de Lucca Júnior W., Farias A.S., de Souza Ribeiro A., Cavaleiro C., Machado S.M.F., Scher R. (2023). Cytotoxic activity of essential oil from Leaves of *Myrcia splendens* against A549 Lung Cancer cells. BMC Complement. Med. Ther..

[49] Pereira R.P., Fachinetto R., de Souza Prestes A., Puntel R.L., Santos da Silva G.N., Heinzmann B.M., Boschetti T.K., Athayde M.L., Bürger M.E., Morel A.F. (2009). Antioxidant Effects of Different Extracts from *Melissa officinalis*, *Matricaria recutita* and *Cymbopogon citratus*. Neurochem. Res..

[50] San Miguel-Chávez R., Soto-Hernandez M., Palma-Tenango M., Garcia-Mateos M. (2017). Phenolic Antioxidant Capacity: A Review of the State of the Art. Phenolic Compounds.

[51] Jaber A., Soukariyeh R., Khalil A., Abdel-Sater F., Cheble E. (2020). Biological Activities of Total Oligomeric Flavonoids Enriched Extracts of *Nicotiana tabacum* from Eight Lebanese Regions. Int. J. Pharm. Sci. Rev. Res..

[52] Barreca D., Laganà G., Leuzzi U., Smeriglio A., Trombetta D., Bellocco E. (2016). Evaluation of the nutraceutical, antioxidant and cytoprotective properties of ripe pistachio (*Pistacia vera* L., variety Bronte) hulls. Food Chem..

[53] Singleton V.L., Orthofer R., Lamuela-Raventós R.M. (1999). [14] Analysis of total phenols and other oxidation substrates and antioxidants by means of folin-ciocalteu reagent. Methods Enzymol..

[54] Awada N., Ayoub A., Jaber A., Ibrahim F., El Ghotmi N., Cheble E. (2023). Evaluation of the Anticancer, Anti-Inflammatory, and Antioxidant Properties of Various Extracts of *Annona squamosa* L.. Pharm. Sci..

[55] Oyaizu M. (1986). Studies on Products of Browning Reactions: Antioxidative Activities of Product of Browning Reaction Prepared from Glucosamine. Jpn. J. Nutr. Diet..

[56] Balouiri M., Sadiki M., Ibnsouda S.K. (2016). Methods for in vitro evaluating antimicrobial activity: A review. J. Pharm. Anal..

[57] Semaan J., El-Hakim S., Ibrahim J.-N., Safi R., Elnar A., El Boustany C. (2020). Comparative effect of sodium butyrate and sodium propionate on proliferation, cell cycle and apoptosis in human breast cancer cells MCF-7. Breast Cancer.

[58] Ibrahim J.-N., El-Hakim S., Semaan J., Ghosn S., El Ayoubi H., Elnar A.A., Tohme N., El Boustany C. (2024). Sodium Butyrate (NaB) and Sodium Propionate (NaP) Reduce Cyclin A2 Expression, Inducing Cell Cycle Arrest and Proliferation Inhibition of Different Breast Cancer Subtypes, Leading to Apoptosis. Biomedicines.

[59] Sousa E.O., Barreto F.S., Rodrigues F.F., Campos A.R., Costa J.G. (2012). Chemical composition of the essential oils of *Lantana camara* L. and *Lantana montevidensis* Briq. and their synergistic antibiotic effects on aminoglycosides. J. Essent. Oil Res..

[60] Walden A.B., Haber W.A., Setzer W.N. (2008). Essential Oil Compositions of Three *Lantana* Species from Monteverde, Costa Rica. Nat. Prod. Commun..

[61] Khan M., Mahmood A., Alkhathlan H.Z. (2016). Characterization of leaves and flowers volatile constituents of *Lantana camara* growing in central region of Saudi Arabia. Arab. J. Chem..

