# Supplementary material for: Evaluation of Antiproliferative Potentials Associated with the Volatile Compounds of Lantana camara Flowers: Selective In Vitro Activity
Source: Molecules. 2024 Nov 18;29(22):5431. doi: 10.3390/molecules29225431 (PMC11597665; doi:10.3390/molecules29225431)
Supplement: Supplementary file 1 [file molecules-29-05431-s001.zip › molecules-3236381-supplementary.pdf]

Supplementary Materials

Evaluation of Antiproliferative Potentials Associated with the Volatile Compounds of *Lantana camara* Flowers: Selective In Vitro Activity

Jennifer El Hajj [1,†], Louna Karam [2,†], Ali Jaber [3,*], Edmond Cheble [1], Elias Akoury [3], Philippe Hussein Kobeissy [2], José-Noel Ibrahim [2] and Ali Yassin [3,*]

1. RDMPN Laboratory, Faculty of Pharmacy, Lebanese University, Beirut BP 14/6573, Lebanon; jenniferhajj0@gmail.com (J.E.H.); edmond.cheble@ul.edu.lb (E.C.)
2. Department of Biological Sciences, School of Arts and Sciences, Lebanese American University, Beirut 1102-2801, Lebanon; louna.karam@lau.edu.lb (L.K.); philippehussein.kobeissy@lau.edu.lb (P.H.K.); josenoel.ibrahim@lau.edu.lb (J.-N.I.)
3. Department of Physical Sciences, School of Arts and Sciences, Lebanese American University, Beirut 1102-2801, Lebanon; elias.akoury@lau.edu.lb

* Correspondence*:* ali.jaber14@lau.edu.lb (A.J.); ali.yassin02@lau.edu.lb (A.Y.)
† These authors contributed equally to this work.

1. Collection of the flowers

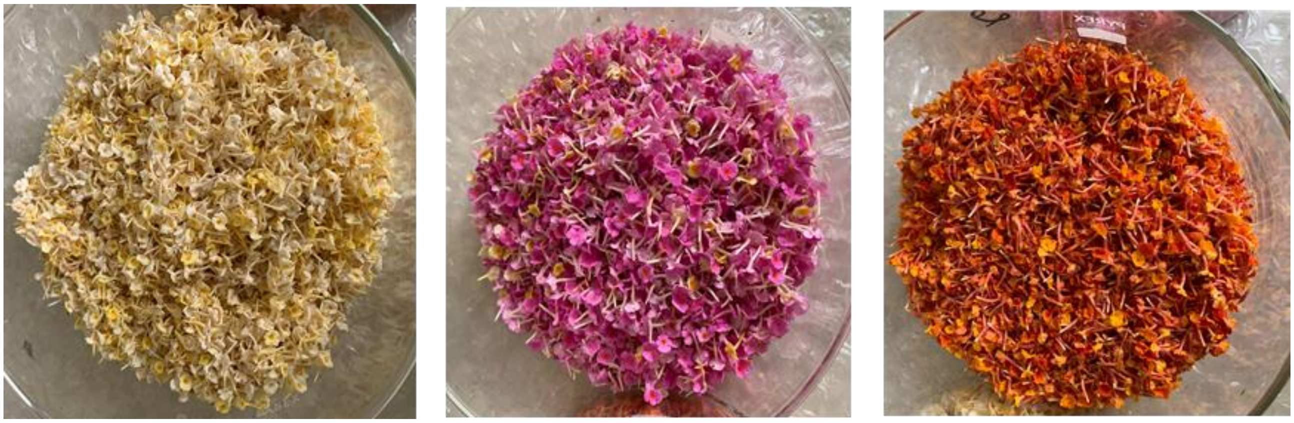

Figure S1*: Petals of the Lantana Camara flowers: White, Pink, and Orange, respectively.*

2. Chemical Profiles of the EOs

Yield of extraction: 0.045%			
**Peak No.**	**Retention time (min)**	**Name**	**Proportion (%)**
1	5,433	p-Cymene	1,5
2	6,364	3-Carene	5,56
3	16,656	5-Trimethyl-6-methylene-cyclohe[illegible]	3,5
4	20,08	**Caryophyllene**	**49,77**
5	21,45	α- humulene	2,71
6	22,633	**(+)-Epibicyclosesquiphellandren[illegible]**	**9,04**
7	23,297	**Bicyclogermacrene**	**21,34**
8	24,465	α-Cubebene	2,62
9	26,379	α-Farnesene	3,95

Table S1: *Main constituents of L. Camara white flowers essential oil (EO1)*

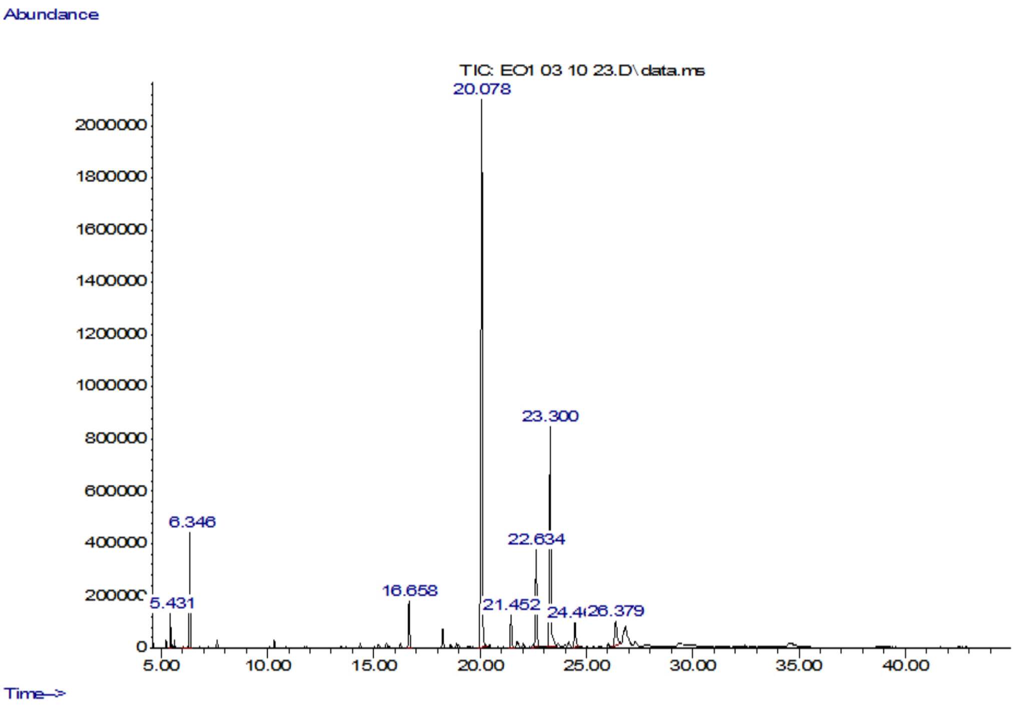

Figure S2: *GC-MS chromatogram of EO1*

Yield of extraction: 0.040%			
**Peak No.**	**Retention time (min)**	**Name**	**Proportion (%)**
1	16,656	Camphene	3,67
2	18,242	Copaene	3,18
3	20,059	**Caryophyllene**	**54,2**
4	22,628	**α-Muurolene**	**12,05**
5	23,287	**γ-Elemene**	**21,37**
6	24,459	α-Cubebene	5,5

***Table S2**: Main constituents of L. Camara pink flowers essential oil (EO2)*

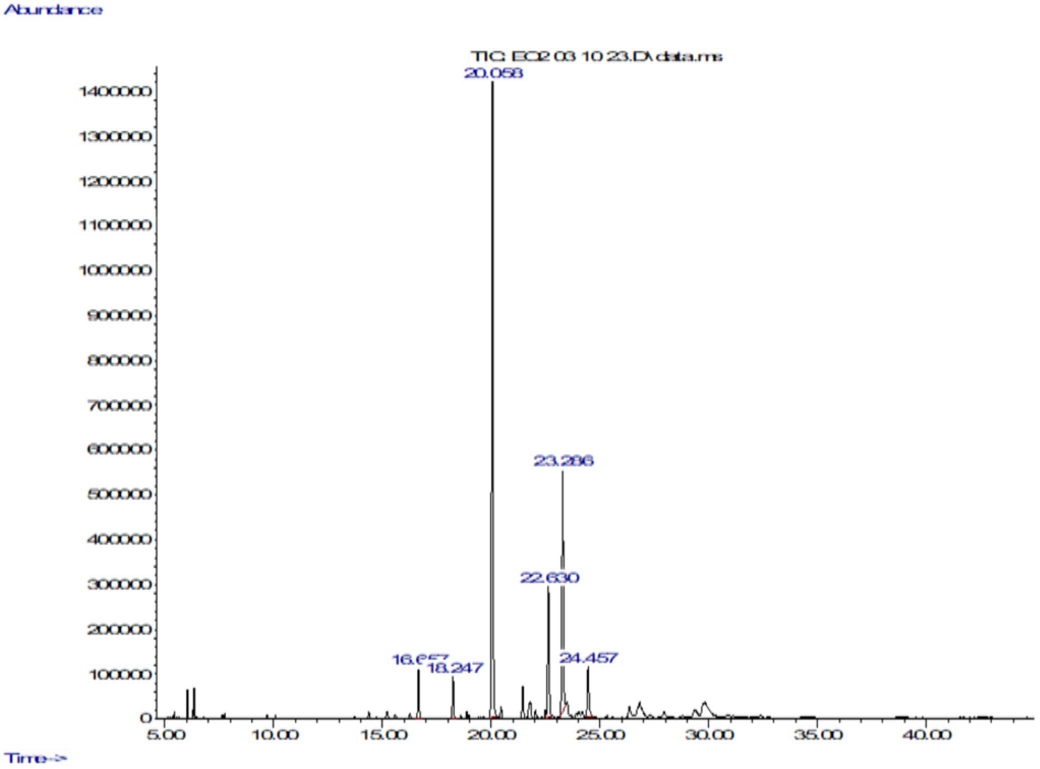

***Figure S3**: GC-MS chromatogram of EO2*

Yield of extraction: 0.047%			
**Peak No.**	**Retention time (min)**	**Name**	**Proportion (%)**
1	6,346	α-Pinene	3,36
2	20,044	**Caryophyllene**	**17,75**
3	21,466	**α-humulene**	**17,42**
4	22,695	**α-*trans*-Bergamotene**	**21,81**
5	22,84	α-Curcumene	4,04
6	23,39	**α-Phellandrene**	**21,48**
7	24,06	Copaene	7,62
8	28,745	α-Farnesene	6,51

***Table S3**: Main constituents of L. Camara orange flowers essential oil (EO3)*

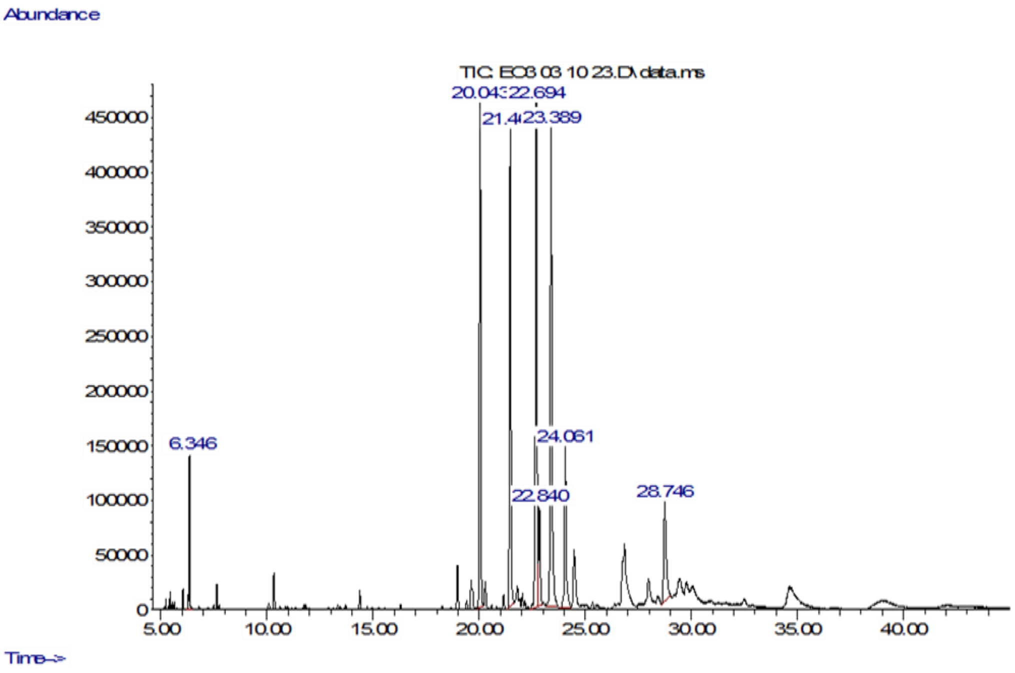

***Figure S4**: GC-MS chromatogram of EO3*

Origin	Major components	Composition (%)	Part of Plant	References
Brazil	Bicyclogermacrene	19,4	Leaves	Ref (1)
	Isocaryophyllene	16,7		
	Valencene	12,9		
	Germacrene D	12,3		
China	Germacrene D	15,85	Leaves & Flowers	Ref (2)
	β-Caryophyllene	12,35		
	α-Humulene	9,31		
	Germacrene-B	6,19		
	1,8-Cineole	4,61		
Costa Rica	Caryophyllene	30,3	Leaves	Ref (3)
	γ-Muurolene	27,2		
	Bicyclogermacrene	24,4		
	β-Elemene	14,2		
Saudi Arabia	Cis-3-hexen-1-ol	11,3	Leaves	Ref (4)
	1-octen-3-ol	8,7		
	Spathulenol	8,6		
	Caryophyllene Oxide	7,5		
	1-hexanol	5,8		
	Caryophyllene oxide	10,6	Flowers	
	β-Caryophyllene	9,7		
	Spathulenol	8,6		
	γ-Cadinene	5,6		
	β-Farnesene	5		
Cairo, Egypt	Davanone	28,59	Leaves	Ref (5)
	Z-Caryophyllene	11,21		

Origin	Major components	Composition (%)	Part of Plant	References
Cairo, Egypt	α-Curcumene	10,26	Leaves	Ref (5)
Cairo, Egypt	β-Copene & Humelene	12,29	Leaves	Ref (5)
	Davanone	23,37	Flowers	
	Caryophyllene	22,96		
	Humulene	14,32		
Côte d'Ivoire	β-Caryophyllene	39,9	Leaves	Ref (6)
	α-Humulene	20,5		
	β-Caryophyllene	36,6	Flowers	
	α-Humulene	19,9		

Table S4*: Main constituents of EOs of Lantana Camara from different geographical origins previously reported in literature.*

Secondary Metabolites	White flower	Pink flower	Orange flower
**Tannins**	-	-	-
**Quinones**	+	+	+
**Carotenoids**	+	+	+
**Terpenoids**	+	+	+
**Anthocyanins**	-	-	-
**Proteins**	-	-	-
**Flavonoids**	+	+	+
**Phenols**	+	+	+
**Alkaloids**	+	+	+
**Saponins**	+	+	+

+ = Present - = Absent

Table S5*: Results of the phytochemical screening of L. Camara flowers.*

3. Analysis of the EOs

1. *TFC and TPC Results*

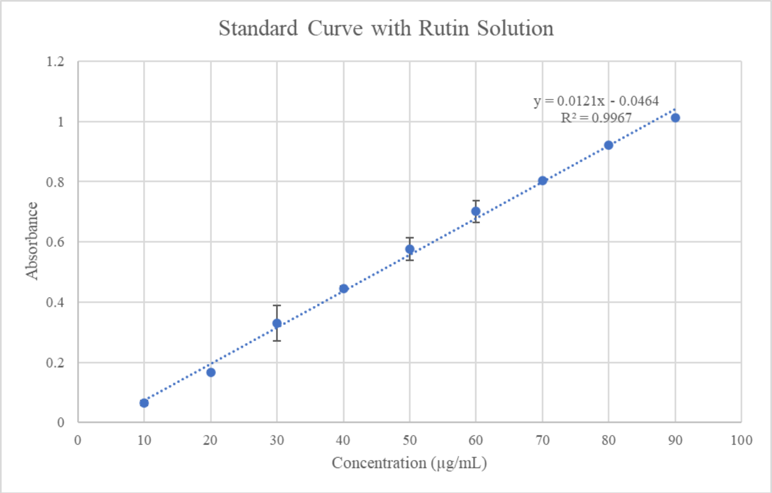

Figure S5: *Variation of the Absorbance in function of the concentration of the Rutin in µg/mL*

Sample	y	x (µg/ml)	C (mg/ml)	mass (g)	TFC (mg of RE/g of extract)
OE1	0,246	24,10	0,0241	0,1	2,41
OE2	0,165	17,45	0,0175	0,08	2,19
OE3	0,388	35,85	0,0359	0,07	5,12

Table S6: *Results of the TFC of the different samples.*

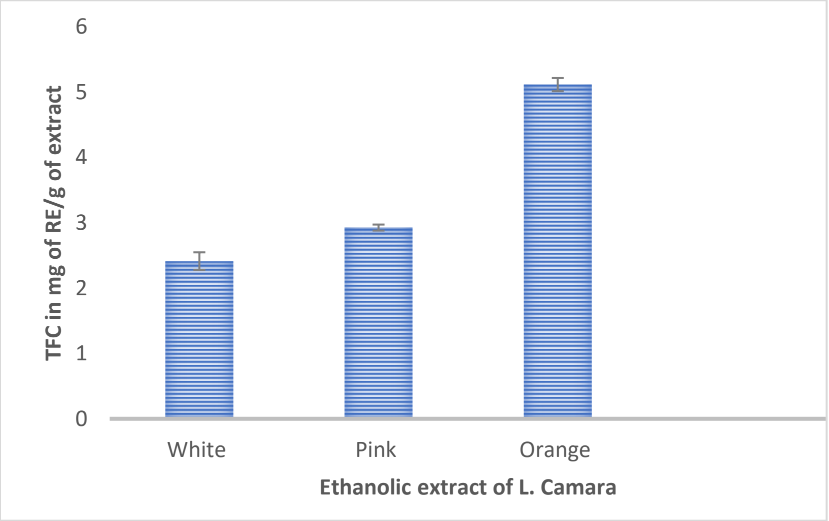

Figure S6: *Comparison of the TFC in different extracts of Lantana Camara*

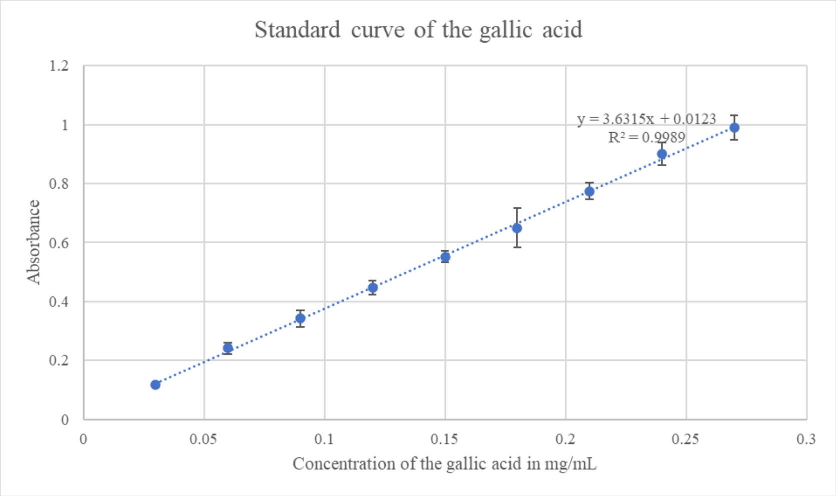

***Figure S7**: Variation of the Absorbance in function of the concentration of Gallic Acid in mg/mL.*

Sample	y	C (mg/ml)	mass (g)	TPC (mg of GAE/g of extract)
OE1	0,62	0,169	0,1	16,8
OE2	0,72	0,197	0,08	24,6
OE3	0,69	0,187	0,07	26,7

***Table S7**: Results of TPC of the different samples.*

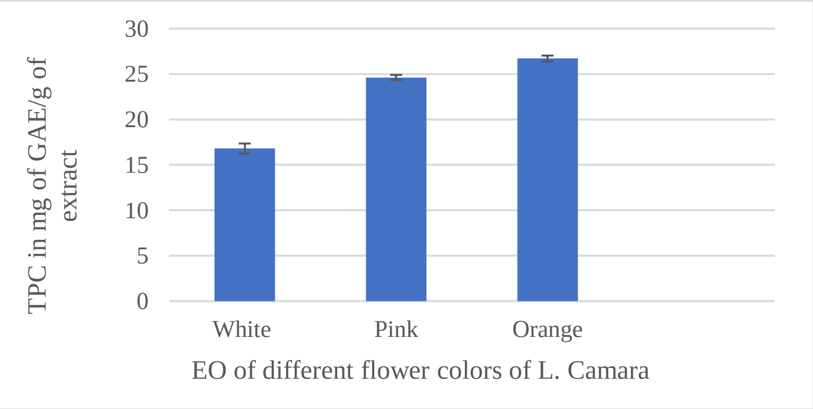

***Figure S8**: Comparison of the TPC in different essential oil extracts of Lantana Camara*

2. DPPH Results

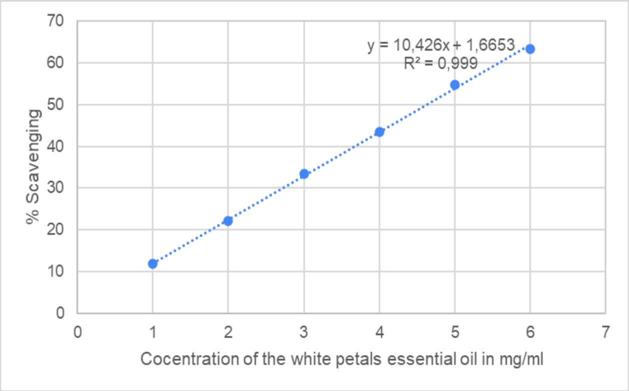

EO1)

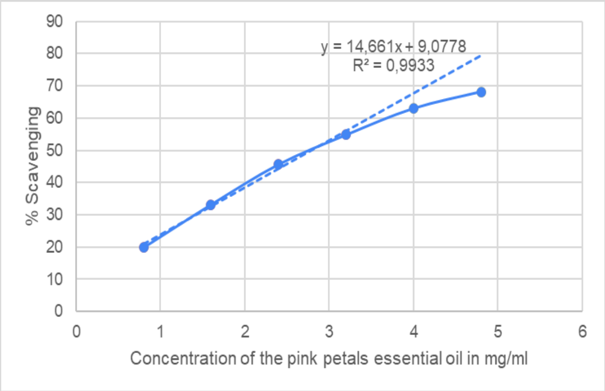

EO2)

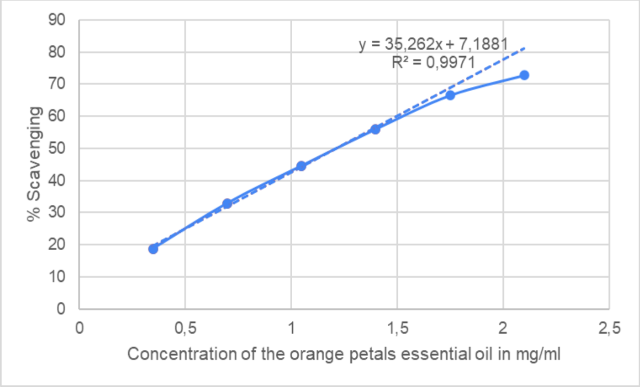

EO3)

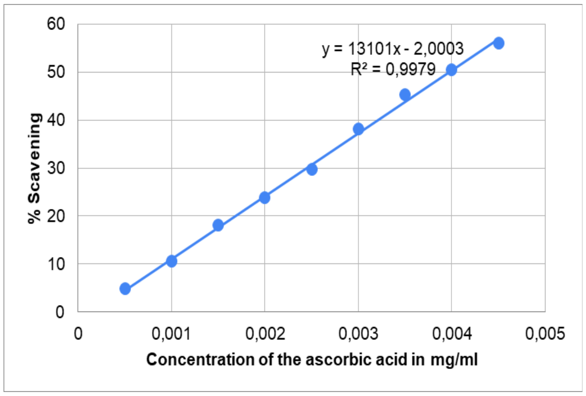

Ascorbic Acid standard)

Figure S9: *Results of the DPPH scavenging assay in the three EOs and the ascorbic acid measuring the % Scavenging in function of its different concentrations in mg/mL.*

Sample	Equation	R^2	IC50 (mg/ml)
EO1	y = 10,426 x + 1,6653	0,999	**4,64**
EO2	y= 14,661 + 9,0778	0,9933	**2,79**
EO3	y= 35,262 x + 7,1881	0,9971	**1.21**
Ascorbic Acid	y = 1301x + 2.0003	0,9979	**0,004**

Table S8*: Results of DPPH Assay of the different samples.*

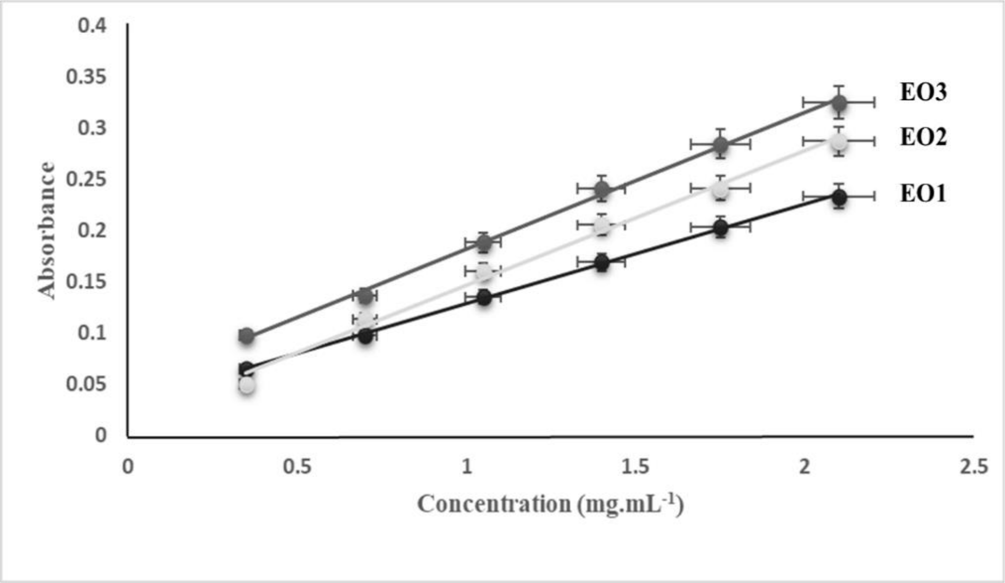

***Figure S10**: Results of the FRAP assay of the three EO samples, measuring absorbance as function of the different concentrations in mg.ml-1.*

3. Pearson Correlation data

Sample	TFC	TPC	DPPH	MCF7	MDAMB-231
**EO1**	2.41	16.8	4.64	0.306	0.3099
**EO2**	2.93	24.63	2.79	0.363	0.7398
**EO3**	5.12	26.71	1.21	0.5927	0.6473

(1) Erlânio O. Sousa, Francisco S. S. Barreto , Fabíola F.G. Rodrigues , Adriana R. Campos & José G.M. **(2012)** Chemical composition of the essential oils of *Lantana camara* L. and *Lantana montevidensis* Briq. and their synergistic antibiotic effects on aminoglycosides, doi: 10.1080/10412905.2012.703494

(2) Sundufu, A.J. and Shoushan, H. **(2004),** Chemical composition of the essential oils of *Lantana camara* L. occurring in south China. doi: 10.1002/ffj.1292

(3) Walden, A. B., Haber, W. A., & Setzer, W. N. (n.d.). **(2008)** Essential Oil Compositions of Three Lantana Species from Monteverde, Costa Rica doi: 10.1177/1934578X0900400123

(4) Khan, M., Mahmood, A., & Alkhathlan, H. Z**. (2016)** Characterization of leaves and flowers volatile constituents of Lantana camara growing in central region of Saudi Arabia. *Arabian Journal of Chemistry*. doi: 10.1016/J.ARABJC.2015.11.005

(5) El Baroty, G. S., Goda, H. M., Khalifa, E. A., Abd, H. H., & Baky, E. **(2014).** Antimicrobial and antioxidant activities of leaves and flowers essential oils of Egyptian Lantana camara L.

(6) Fatimata Nea, Didjour Albert Kambiré, Manon Genva, Evelyne Amenan Tanoh, Esse Leon Wognin, et al. (**2020**), Composition, Seasonal Variation, and Biological Activities of Lantana camara Essential Oils from Côte d’Ivoire. Molecules, doi : 10.3390/molecules25102400
